# BRIDGING THE DIGITAL DIVIDE: EXPLORING DETERMINANTS OF TELEHEALTH UTILIZATION AMONG US OLDER ADULTS

**DOI:** 10.1093/geroni/igae098.2895

**Published:** 2024-12-31

**Authors:** Misun Hwang, Philip Veliz, Yun Jiang

**Affiliations:** University of Michigan, Ann Arbor, Michigan, United States; University of Michigan, Ann Arbor, Michigan, United States; University of Michigan, Ann Arbor, Michigan, United States

## Abstract

The growing older population emphasizes the need for effective integration into the digital health ecosystem. Multiple factors can affect technology adoption among older adults; however, few studies have comprehensively examined their use of telehealth. This study aimed to explore determinants of telehealth utilization among older adults based on the Senior Technology Acceptance Model. Logistic regression was conducted to analyze a nationally representative survey of 6,327 community-dwelling older adults (≥65 years) from the 2022 National Health and Aging Trend Study (NHATS). Telehealth utilization was defined as communicating with health professionals online to manage health. Findings indicated that 46.1% of respondents reported telehealth utilization last year. Respondents who were older, non-white, living in non-metropolitan areas, had lower levels of education and income, or lacked access to technologies were less likely to utilize telehealth. Additionally, poor cognitive function and a lack of social networks or social participation were associated with the non-use of telehealth. However, poorer health status and multiple comorbidities, including cancer (OR=1.47, 95% CI=1.12-1.92) and anxiety (OR=1.56, 95% CI=1.10-2.26), were associated with greater odds of telehealth utilization. Gender, marital status, sensory impairment, limitation of strength or movement, instrumental activities of daily living, and depression were not associated with telehealth utilization. This study confirms the digital divide among vulnerable older adults in the US. Although they have an increased need for telehealth services with their health conditions, they might not fully benefit from telehealth. There is an urgent need to improve digital inclusion and advance health equity in this population.

